# Dispensation of outpatient hospital medicines by hospital only versus hospital-community pharmacies collaboration: a cross-sectional study and survey of patient’s satisfaction

**DOI:** 10.3389/fpubh.2024.1335265

**Published:** 2024-05-03

**Authors:** Olivia Ferrández, Santiago Grau, Elena Colominas-González, María Eugenia Navarrete-Rouco, Nuria Carballo-Martínez, Marta De Antonio-Cuscó, Xènia Fernández-Sala, Laura Rio-No, Oscar Fando Romera, Maria Berzosa Malagon, Sergio Pineda Rodriguez, Noelia Torres Rius, Xavier Duran-Jordà, Cristina Rodríguez-Caba, Jordi Casas-Sánchez, Félix Caro Herranz, Caridad Pontes-García

**Affiliations:** ^1^Service of Hospital Pharmacy, Hospital del Mar, Barcelona, Spain; ^2^Facultat de Medicina i Ciències de la Vida. Universitat Pompeu Fabra, Barcelona, Spain; ^3^Department of Statistics, Institut Hospital del Mar d’Investigacions Mèdiques (IMIM), Barcelona, Spain; ^4^Col·legi Oficial de Farmacèutics de Barcelona, Barcelona, Spain; ^5^Department of Informatics, Hospital del Mar, Barcelona, Spain; ^6^Gerència del Medicament del Servei Català de Salut, Barcelona, Spain

**Keywords:** community pharmacy, outpatient hospital medications, patient satisfaction, pharmaceutical care, hospital pharmacy

## Abstract

**Goal:**

To describe the experience of a dispensing model of outpatient hospital medicines (OHM) via collaboration of hospital and community pharmacies, and to explore patient satisfaction with the strategy as compared with the hospital pharmacy only service.

**Background:**

Patient satisfaction is an important component of the quality of health care.

**Study:**

A new model of dispensing OHM was conducted in the Outpatients Unit of the Service of Hospital Pharmacy of Hospital del Mar, in Barcelona, Spain. Participants were patients on stable chronic treatment with clinical or social fragility, immunocompromised patients, and those whose residence was located at a distance from the hospital that justified drug delivery through the community pharmacy. A cross sectional study was done using an *ad hoc* 14-item questionnaire collecting demographic data, duration of treatment, usual mode of collecting medication, and the degree of satisfaction regarding waiting time for the collection of medication, attention received by professionals, information received on treatment, and confidentiality.

**Results:**

The study population included a total of 4,057 patients (66.8% men) with a mean age of 53 (15.5) years, of whom 1,286 responded, with a response rate of 31.7%. Variables significantly associated with response to the survey were age over 44 years, particularly the age segment of 55–64 years (odds ratio [OR] 2.51) and receiving OHM via the community pharmacy (OR 12.76). Patients in the community pharmacy group (*n* = 927) as compared with those in the hospital pharmacy group (*n* = 359) showed significantly higher percentages of ‘satisfied’ and ‘very satisfied’ (*p* < 0.001) in the waiting time for the collection of OHM (88.1% vs. 66%), attention received by professionals (92.5% vs. 86.1%), and information received on treatment (79.4% vs. 77.4%). In relation to confidentiality, results obtained were similar in both pharmacy settings.

**Conclusion:**

Dispensing OHM through the community pharmacy was a strategy associated with greater patient satisfaction as compared with OHM collection at the hospital pharmacy service, with greater accessibility, mainly due to close distance to the patient’s home. The participation of community pharmacists could further optimize the care received by patients undergoing OHM treatment.

## 1 Introduction

Patients undergoing treatment with outpatient hospital medicines (OHM) should regularly visit the hospital pharmacy to collect their medication. OHM are specific medicines that by law require to be dispensed by hospital pharmacy services in Spain (1). The outpatient units of the hospital pharmacy services developed specialized pharmaceutical care for outpatients who require treatment with hospital-dispensed drugs, including validation of the prescription, comprehensive monitoring of treatment, and direct information to patients about correct administration of the medication, how to improve compliance, and how to manage possible interactions, adverse drug events, or toxicities. Consistently providing high-quality pharmaceutical services and medication management, particularly in patients with chronic conditions, has been shown to be a cornerstone of patient satisfaction and an essential factor to ensure adherence with medication (2–4). Adherence to chronic treatments is challenging and has stimulated health care providers to devise differentiated service delivery models to decentralize chronic medicine distribution at health care facilities (5–7). On the other hand, strategies to facilitate the processes involved in obtaining medications are important to maintain patients’ key outcomes.

Additionally, the public health emergency situation caused by COVID-19 pandemic forced the establishment of immediate unconventional strategies with the aim of dealing with an unprecedented global health crisis (8), and was also a challenge in the dispensing of OHM. Due to this situation, alternatives through home delivery models or active involvement of community pharmacies offered convenient options as drug dispensing systems, primarily ensuring treatment continuity (9, 10). Community pharmacies were allowed to continue operating during the pandemic, and played a key role as strategically positioned resources for multiple services. These includes decongestion of public health facilities, and management of patients with chronic diseases (11). In this context, CatSalut, which is the body that ensures quality public health care for the citizens of Catalonia - an autonomous community in Northeastern Spain, 7,722,203 inhabitants, 2022 census- set a protocol with the Official College of Pharmacists of Barcelona. The protocol described procedures for the dispensing and delivery of OHM via community pharmacies. Also, Catsalut and the Department of Health issued legal coverage for the activity (12, 13). One the purposes of the action was to diminish the density of patients accessing the hospital during the COVID-19 lock-down, and also to reduce the potential transmission of SARS-CoV-2 infection while in pandemic period. Moreover, this mode of dispensing of OHM through nearby community pharmacies increases the ease of treatment accessibility and prevents the necessity of traveling to the hospital, and has continued after the COVID-19 period.

Different studies especially focused on the administration of antiretroviral therapy (ART) have shown the feasibility, acceptability, positive clinical results and high adherence rates of dispensing models based on the community pharmacy (14, 15). However, added value in terms of patient experience, as measured through patient satisfaction concerning dispensing of OHM by community pharmacies, has not been fully evaluated previously. In this cross-sectional study, our objective was to describe our experience and to explore patient satisfaction to which the strategy of OHM dispensing model via the community pharmacy was offered compared to the traditional approach of drug dispensing at the hospital pharmacy.

## 2 Materials and methods

### 2.1 Study design and setting

A cross-sectional study was conducted in Outpatients Unit of the Service of Hospital Pharmacy of Hospital del Mar, in Barcelona, Spain. The hospital catchment area includes approximately 300,000 people living in two urban districts of the city of Barcelona (1,639,981 inhabitants, census 2022).

The study protocol was approved by the Clinical Research Ethics Committee of Parc de Salut Mar (code CEImPSMAR 2020-9,608, approval date March 1, 2021). Oral informed consent was obtained from all participants.

### 2.2 Participants

The study population included patients with medical conditions of different specialties who regularly received OHM dispensed by the Service of Hospital Pharmacy of Hospital de Mar. Candidates to OHM dispensing via community pharmacies met the following criteria, according to the agreed protocol (13): patients on stable chronic treatment with clinical or social fragility, immunocompromised patients, and those whose residence was located at a distance from the hospital that justified drug delivery through the community pharmacy.

### 2.3 Remote OHM procedures

Remote OHM dispensing shared with community pharmacies was introduced in the hospital in March 2020, shortly after initiation of COVID-19 pandemic wave. Eligible patients were selected by staff pharmacists (S.G.C., O.F.Q.). Once the patient was identified, he/she was offered the possibility of benefiting from the circuit. In case the patient agreed, a community pharmacy was chosen by the patient to pick up his/her OHM, generally a pharmacy close to the patient’s home. To this purpose, the College of Pharmacists of Barcelona together with members of the Service of Hospital Pharmacy of Hospital del Mar, designed a computer application to optimize the management of delivery of OHM from the hospital pharmacy to community pharmacies.

Every working day, the hospital pharmacist reviewed a list of patients who would require dispensing their OHM treatment. Prior to preparing the medication for dispatch to the community pharmacy, the following aspects were checked: (a) that neither changes in the prescription, modification, and/or interruption of the treatment for any reason had been made; (b) that the patient had attended his/her scheduled appointment with the doctor; and (c) that the patient had collected the OHM on time previously. Once these points had been confirmed, the treatment was marked as ‘reviewed’ on the electronic platform, at which time the pharmacy technician could proceed with its preparation. When OHM treatments for all the patients for that day have been prepared, medications were distributed to the different community pharmacies by an authorized pharmaceutical distributor.

### 2.4 Survey

Between January and June, 2022, a survey was sent via SMS to the mobile phones of the patients undergoing treatment with OHM who had consented, both those who received it through community pharmacies and those who continued to attend the Outpatient Unit of the Service of Hospital Pharmacy.

In the absence of validated surveys in the area of the objective of this study, the items included in the questionnaire were agreed upon with experts in the area of patients undergoing OHM treatment, including physicians and pharmacists.

An *ad hoc* 14-item questionnaire was designed to collect demographic data, duration of treatment, usual mode of collecting medication, and the degree of satisfaction regarding waiting time for the collection of medication, attention received by professionals, information received on treatment, and confidentiality, which were rated on a 5-point Likert scale (very satisfied, satisfied, neither satisfied nor dissatisfied, dissatisfied, very dissatisfied). The same questions were addressed to patients who picked up their OHM at the hospital and those who collected it from the community pharmacy.

Patients did not receive any incentives for their participation in the survey.

The details of the questionnaire are shown in the Supplementary material.

### 2.5 Study endpoints

The primary endpoint was the patients’ overall degree satisfaction associated with dispensing OHM, compared between patients who came to the hospital pharmacy or those collecting it in the community pharmacies. Secondary endpoints included identification of variables associated with response to the survey and with satisfaction with each OHM dispensing mode.

### 2.6 Data collection

For each patient the following data were collected: gender; age group (≤ 44 years, between 45 and 54 years, between 55 and 64 years, and ≥ 65 years); pharmacological group of OHM (dermatology, rheumatology, nephrology, and ART); dispensing mode (hospital vs. community pharmacy); and responses to each of the items of the questionnaire.

### 2.7 Statistical analysis

Categorical variables are expressed as frequencies and percentages, and continuous variables as mean and standard deviation (SD) or median and interquartile range (IQR) (25th-75th percentile). The chi-square test or the Fisher’s exact test was used for the comparison of categorical variables, and the Student’s *t* test for the comparison of quantitative variables. Binary or ordinal regression analysis was used to assess differences in the distribution of study variables between the OHM dispensing mode (hospital vs. community pharmacy). Multivariable analysis was used to assess factors independently associated with patients’ satisfaction, using estimated odds ratio (OR) and 95% confidence intervals (CIs). Statistical significance was set at *p* < 0.05. All statistical data were analyzed using STATA software (version 15.1) (StataCorp LLC, College Station, TX, United States).

## 3 Results

**Figure 1:**
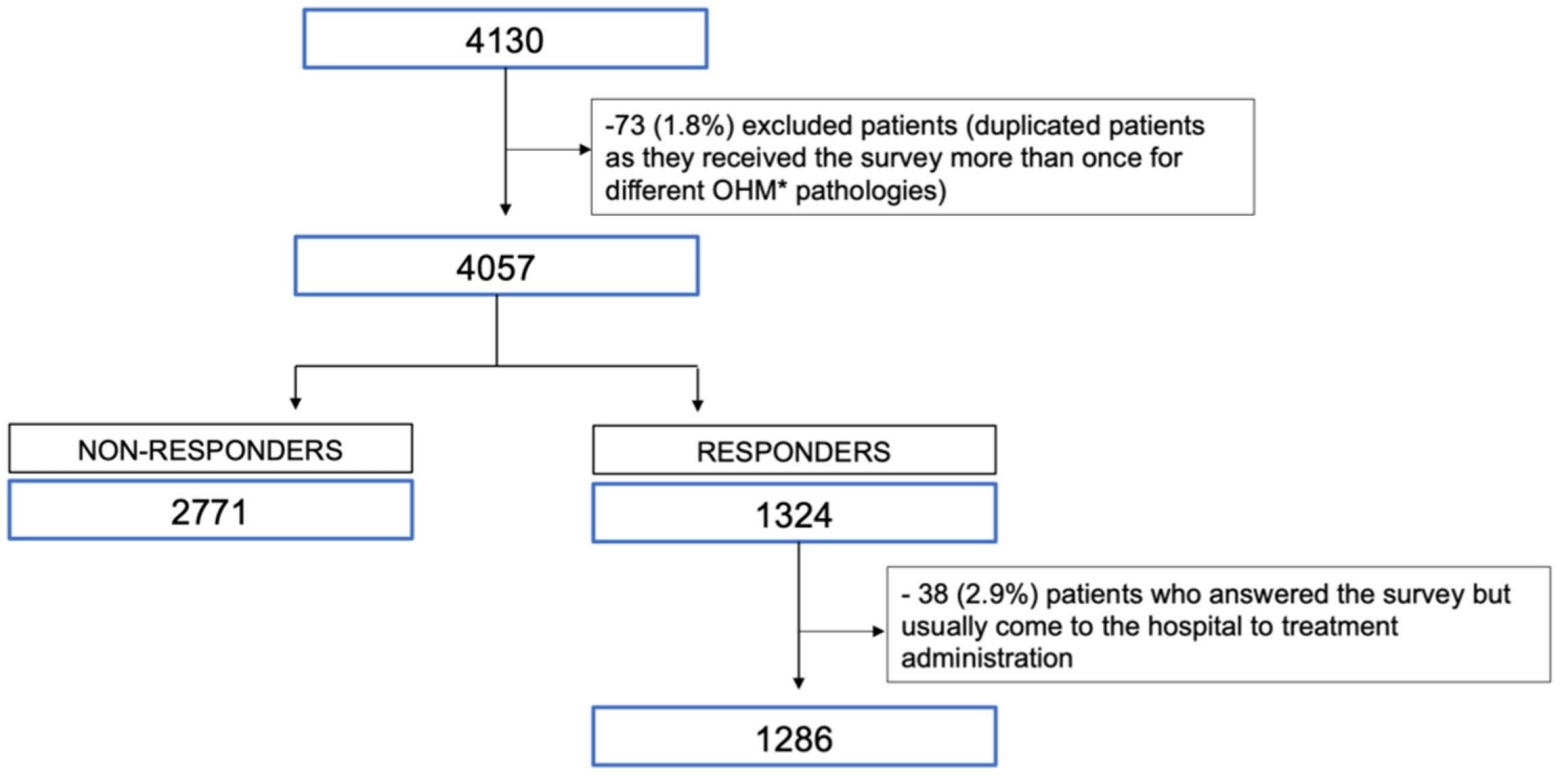
Flow diagram for patient enrollment. *OHM: outpatient hospital medicines.

The study population included a total of 4,057 patients (66.8% men, mean age of 53 (15.5) years) who met the inclusion criteria and received the study questionnaire. Of those, 1,286 responded, with a response rate of 31.7%. Figure 1 shows a flow diagram for patient enrolment.

As shown in Table 1, there were statistically significant differences in the characteristics of responders and non-responders. Responders were younger, and the percentage of males was lower than among non-responders. The percentage of responders who picked up their OHM at community pharmacies was higher than in non-responders (72.1% vs. 12.2%, *p* < 0.001). Antiretroviral drugs were the most commonly dispensed medication followed by rheumatology drugs. The percentages of patients on ART and using nephrology drugs were higher among non-responders, whereas the percentages of patients using dermatology and rheumatology drugs were higher among responders.

**Table 1:** Differences between responders and non-responders to the study survey.

Variables	All patients (*n* = 4,057)	Responders (*n* = 1,286)	Non-responders (*n* = 2,771)	*p*-value
Gender				0.025
Male	2,711 (66.8)	828 (64.4)	1,883 (67.9)	
Female	1,346 (33.2)	458 (35.6)	888 (32.0)	
Age, years, mean (SD)	53.0 (15.5)	54.3 (13.4)	52.4 (16.4)	< 0.001
< 44 years	1,251 (30.8)	289 (22.5)	962 (34.7)	< 0.001
45–54 years	998 (24.6)	353 (27.4)	645 (23.3)	
55–64 years	922 (22.7)	378 (29.4)	544 (19.6)	
≥ 65 years	886 (21.8)	266 (20.7)	620 (22.4)	
Dispensing pharmacy				< 0.001
Hospital	2,843 (70.1)	359 (27.9)	2,434 (87.8)	
Community	1,213 (29.9)	927 (72.1)	337 (12.2)	
Pharmacological group				< 0.001
Dermatology	273 (6.7)	107 (8.3)	166 (6.0)	
Nephrology	399 (9.8)	57 (4.4)	342 (12.3)	
Rheumatology	778 (19.2)	357 (27.8)	421 (15.2)	
Antiretroviral	2,116 (52.2)	601 (46.7)	1,515 (54.7)	
Other	491 (12.1)	164 (12.7)	327 (11.8)	

SD, standard deviation. Data expressed as frequencies and percentages in parenthesis unless otherwise stated.

In the multivariable analysis (Table 2), response to the survey was significantly more frequent for patients aged over 44 years, particularly the age segment of 55–64 years (OR 2.51), and those receiving OHM via the community pharmacy (OR 12.76). On the other hand, treatment with nephrology drugs were associated with a lower probability of response as compared with dermatology drugs.

**Table 2:** Variables associated with response to the survey.

Variables	Odds ratio (95% confidence interval)	*p*-value
*Gender*		
Female	1.00 (reference)	
Male	1.06 (0.88 to 1.27)	0.556
*Age, years*		
< 44 years	1.00 (reference)	
45–54 years	1.93 (1.55 to 2.42)	< 0.001
55–64 years	2.51 (2.0 to 3.15)	< 0.001
≥ 65 years	1.75 (1.35 to 2.27)	< 0.001
*Dispensing pharmacy*		
Hospital	1.0 (reference)	
Community	15.15 (12.76 to 17.99)	< 0.001
*Pharmacological group*		
Dermatology	1.0 (reference)	
Nephrology	0.43 (0.27 to 0.68)	< 0.001
Rheumatology	1.14 (0.80 to 1.62)	0.482
Antiretroviral	0.87 (0.62 to 1.20)	0.396
Other	0.63 (0.43 to 0.93)	0.020

In patients who responded to the survey (Table 3), there was a significantly higher percentage of men in the hospital pharmacy group (73.8%) than in the community pharmacy group (60.7%) (*p* < 0.001). Antiretroviral medications were more frequent in the hospital pharmacy group (64.3% vs. 39.9%), whereas rheumatology drugs were more common in the community pharmacy group (33.5% vs. 12.8%) (*p* < 0.001). Also, the duration of time since when they were collecting OHM in any model was over 5 years in 61.3% of patients included in the hospital pharmacy group and 57.7% in the community pharmacy group, and less than 5 years, in 38.7 and 42.2%, respectively.

**Table 3:** Distribution of variables and satisfaction among responders according to the site of delivering of outpatient hospital medicines (OHM).

Variables	All responders (*n* = 1,286)	Community pharmacy (*n* = 927)	Hospital pharmacy (*n* = 359)	*p-*value
Gender				< 0.001
Male	828 (64.4)	563 (60.7)	265 (73.8)	
Female	458 (35.6)	364 (39.3)	94 (26.2)	
Age, years, mean (SD)	54.3 (13.4)	54.3 (13.5)	54.1 (13.1)	0.465
< 44 years	289 (22.5)	203 (21.9)	86 (23.9)	0.432
45–54 years	353 (27.4)	254 (27.4)	99 (27.6)	
55–64 years	378 (29.4)	268 (28.9)	110 (30.6)	
≥ 65 years	266 (20.7)	202 (21.8)	64 (17.8)	
Pharmacological group				< 0.001
Dermatology	107 (8.3)	72 (7.8)	35 (9.7)	
Nephrology	57 (4.4)	27 (2.9)	30 (8.3)	
Rheumatology	357 (27.8)	311 (33.5)	46 (12.8)	
Antiretroviral	601 (46.7)	370 (39.9)	231 (64.3)	
Other	164 (12.7)	147 (15.8)	17 (4.7)	
OHM pick up time, years				0.003
< 1	109 (8.5)	67 (7.2)	42 (11.7)	
1–5	422 (32.8)	325 (35.0)	97 (27.0)	
6–10	276 (21.5)	205 (22.1)	71 (19.8)	
> 10	479 (37.2)	330 (35.6)	149 (41.5)	
Waiting time for the collection of OHM				< 0.001
Very dissatisfied	14 (1.1)	9 (1.0)	5 (1.4)	
Dissatisfied	16 (1.2)	9 (1.0)	7 (1.9)	
Neither satisfied nor dissatisfied	202 (15.7)	92 (9.9)	110 (30.6)	
Satisfied	380 (29.5)	265 (28.6)	115 (32.0)	
Very satisfied	674 (52.4)	552 (59.5)	122 (34.0)	
Attention received by professionals				< 0.001
Very dissatisfied	4 (0.3)	3 (0.3)	1 (0.3)	
Dissatisfied	10 (0.8)	5 (0.5)	5 (1.4)	
Neither satisfied nor dissatisfied	105 (8.2)	61 (6.6)	44 (12.2)	
Satisfied	305 (23.7)	199 (21.5)	106 (29.5)	
Very satisfied	862 (67.0)	659 (71.1)	203 (56.5)	
Information received on treatment				0.002
Very dissatisfied	12 (0.9)	5 (0.5)	7 (1.9)	
Dissatisfied	17 (1.3)	11 (1.2)	6 (1.7)	
Neither satisfied nor dissatisfied	243 (18.9)	175 (18.9)	68 (18.9)	
Satisfied	400 (31.1)	267 (28.8)	133 (37.0)	
Very satisfied	614 (47.7)	469 (50.6)	145 (40.4)	
Confidentiality				0.055
Very dissatisfied	11 (0.8)	7 (0.7)	4 (1.1)	
Dissatisfied	27 (2.1)	18 (1.9)	9 (2.5)	
Neither satisfied nor dissatisfied	225 (17.5)	159 (17.1)	66 (18.4)	
Satisfied	383 (29.8)	259 (27.9)	124 (34.5)	
Very satisfied	640 (49.8)	484 (52.2)	156 (43.4)	

SD, standard deviation. Data expressed as frequencies and percentages in parenthesis unless otherwise stated.

As compared with those in the hospital pharmacy group, patients in the community pharmacy group showed significantly higher percentages of “very satisfied” (*p* < 0.001) in the waiting time for the collection of OHM (59.5% vs. 34%), attention received by professionals (71.1% vs. 56.5%), and information received on treatment (50.6% vs. 40.4%) (Table 3). In relation to confidentiality, results obtained were similar in both pharmacy settings. No differences were observed in the reported satisfaction regarding confidentiality.

Variables independently associated with satisfaction are shown in Table 4. The degree of satisfaction was significantly higher (*p* < 0.001) for the community pharmacy setting in all items of the questionnaire, except for confidentiality, which did not show differences. The waiting time to collect the OHM was the item with stronger association to satisfaction (OR 3.09, 95% CI 2.41–3.95). Men also showed a significantly higher degree of satisfaction than women in the waiting time to collect medication (OR 1.31, 95% CI 1.03–1.66, *p* = 0.027). Also, the age segment of 45–54 years was associated with a higher degree of satisfaction than subjects in the older groups. Treatment with nephrology drugs and ART showed a lower degree of satisfaction with the attention received by professionals, and treatment with ART was also associated with a lower degree of satisfaction regarding confidentiality.

**Table 4:** Variables associated with patient satisfaction.

Variables	Waiting time for the collection of OHM OR (95% CI), *p*-value	Attention received by professionals OR (95% CI), *p*-value	Information received on treatment OR (95% CI), *p*-value	Confidentiality OR (95% CI), *p*-value
*Gender*				
Female	1.0 (ref.)	1.0 (ref.)	1.0 (ref.)	1.0 (ref.)
Male	1.31 (1.03–1.66), **0.027**	1.12 (0.86–1.45), 0.407	0.97 (0.77–1.22), 0.806	1.11 (0.88–1.40), 0.381
*Age, years*				
< 44 years	1.0 (ref.)	1.0 (ref.)	1.0 (ref.)	1.0 (ref.)
45–54 years	1.44 (1.05–1.99), **0.026**	1.10 (0.77–1.57), 0.595	0.90 (0.66–1.23), 0.511	1.30 (0.96–1.77), 0.095
55–64 years	0.88 (0.64–1.20), 0.408	0.78 (0.56–1.10), 0.163	0.84 (0.62–1.13), 0.247	1.11 (0.82–1.50). 0.505
≥ 65 years	0.80 (0.57–1.13), 0.199	0.66 (0.45–0.96), 0.029	0.70 (0.50–0.99), **0.042**	1.08 (0.77–1.51), 0.653
*Pharmacological group*				
Dermatology	1.0 (ref.)	1.0 (ref.)	1.0 (ref.)	1.0 (ref.)
Nephrology	0.56 (0.30–1.05), 0.071	0.49 (0.25–0.98), **0.044**	0.74 (0.40–1.36), 0.329	0.59 (0.32–1.09), 0.094
Rheumatology	0.82 (0.53–1.28), 0.385	0.68 (0.41–1.12), 0.131	0.73 (0.47–1.11), 0.144	0.81 (0.53–1.26), 0.355
Antiretroviral	0.71 (0.46–1.10), 0.122	0.56 (0.34–0.91), **0.019**	0.66 (0.44–1.00), 0.052	0.57 (0.37–0.87), **0.010**
Other	0.91 (0.55–1.51), 0.719	0.91 (0.51–1.61), 0.747	0.68 (0.42–1.09), 0.110	0.75 (0.46–1.20)0.0.231
*Dispensing pharmacy*				
Hospital	1.0 (ref.)	1.0 (ref.)	1.0 (ref.)	1.0 (ref.)
Community	3.09 (2.41–3.95), <**0.001**	1.81 (1.40–2.36), **<0.001**	1.37 (1.08–1.74), **0.010**	1.22 (0.96–1.55), 0.097
*OHM pick up time, years*				
< 1	1.0 (ref.)	1.0 (ref.)	1.0 (ref.)	1.0 (ref.)
1–5	1.17 (0.78–1.77), 0.488	1.28 (0.82–2.00), 0.282	0.88 (0.59–1.32), 0.552	1.16 (0.77–1.74), 0.486
6–10	1.09 (0.70–1.70), 0.692	1.28 (0.79–2.07), 0.318	1.00 (0.64–1.54), 0.989	1.03 (0.66–1.59), 0.901
> 10	1.05 (0.68–1.62), 0.824	1.28 (0.79–2.07), 0.373	0.74 (0.48–1.12), 0.153	1.09 (0.71–1.66), 0.707

OHM, outpatient hospital medicines; OR, odds ratio; CI, confidence interval; ref: reference.

## 4 Discussion

Improvement of coordination of interprofessional actions and initiatives, as well as using all available resources as effectively as possible, has become a consistent goal to optimize health care system outcomes (16). Hospital pharmacists are key to complete the patient’s therapeutic management by connecting the patient (or caregivers) with physicians and other members of a patient’s health care team, ensuring that the highly complex hospital-based treatments are delivered, informed, understood and properly used by the patient, both in the inpatient and outpatient setting. On the other hand, community pharmacists, in addition to their role as dispensers of retail and prescription outpatient medication, are also highly accessible points of therapeutic information and healthcare assistance that may substantially impact on patient care (17) and satisfaction (18–21).

Patient satisfaction has been related to prior patient exposure to services and their level of expectation (22). Although the public is greatly satisfied with community pharmacists’ professionalism and pharmaceutical services, customers’ opinions have been shown to be influenced by pharmacists’ availability and knowledge, pharmacy service promptness, pharmacy location, waiting area, medication knowledge, and counseling (23). However, studies focused on the comparison of patient satisfaction between OHM delivered exclusively by hospital pharmacy services or shared models in collaboration with community pharmacies have not been published.

In the present survey, a simple 14-item questionnaire addressed four aspects related to satisfaction with the pharmaceutical services related to dispensing and delivering OHM. The survey was delivered to 4,057 patients, with a response rate of 31.7%, which can be considered as acceptable. Studies on patient satisfaction carried out in the outpatient setting have shown highly variable response rates (from 16.5 to 86.9%) probably related to the method used for the survey (24–26). In our study, the response rate in patients older than 45 years (69.2%) was more than double that in those younger than 45 years (30.8%). In a study of 2,762 outpatients of a department of orthopedic surgery in an academic center in Salt Lake City (UT, United States) who completed a Press-Ganey patient satisfaction survey, advancing age increased the odds of responding (adjusted OR 3.39 for ≥65 years vs. 18–29 years reference category) (26). On the other hand, the higher rate of response among patients in the community pharmacy group (72.1%) as compared with patients in the hospital pharmacy group (27.9%) may be related to the higher degree of satisfaction associated with the strategy of collaborative dispensing of OHM through the community pharmacy setting.

Dispensing OHM though a collaborative model involving the community pharmacy, as compared to the model using the outpatient unit of the hospital pharmacy service only, resulted in a significantly higher degree of satisfaction in three of the four items evaluated (waiting time, quality of attention received by professionals and information about treatment). Despite the fact that a higher degree of satisfaction in the confidentiality variable was also found in the community pharmacy setting, this association was not significantly different between the models. Patients receiving ART showed a significantly lower degree of satisfaction related to confidentiality in general, which may be related to the stigmatized identity of these patients despite impressive improvements in HIV care (27). These findings highlight the importance of redesigning outpatient care spaces both in the hospital and the community, considering the preservation of patient confidentiality during their stay in the pharmacy, ensuring privacy when interacting with pharmacy personnel.

Clearly excessive and prolonged patient waiting time for collecting medication undermines pharmacy efficiency and impacts the perceived quality of services. In a literature search, Alam et al. (28) have identified methods and technological advancements that have been successfully employed to reduce patient waiting time, such as automated pharmacy devices/machines for quick and accurate filling and dispensing, automated queuing technology, or tele-pharmacy. (29) describe a project conducted in the outpatient pharmacy of a cancer center in Amman (Jordan) based on the identification of current conditions and causes of delay in waiting time, followed by formation of a multidisciplinary team and implementation of a problem solving method based on lean management. This strategy was associated with a significant decrease of the waiting time for prescription of 3 or more medications together with a significant increase in patient satisfaction. In another study, Bleustein et al. (24) collected data regarding patient satisfaction from a sample of 11,352 survey responses returned by patients over the course of 1 year across all 44 ambulatory clinics within a large academic medical center in New York. In a multivariate regression model, a waiting time of 10 min resulted in about a 77% chance of receiving the highest satisfaction score. Moreover, as the time of waiting was increased, the chance of obtaining the highest score decreased. Interestingly, this study showed that increased waiting times also affected perceptions of information, instructions, and the overall treatment provided by physicians and other caregivers. In our study, the collaborative model showed a significant and relevant difference in the satisfaction with the waiting time, that is likely reflecting one of the key advantages of the model, a higher availability with lower waiting times of the community pharmacies. Also, questions on waiting time may also reflect a shorter time required to travel to a nearby community pharmacy, as compared to the hospital pharmacy.

In relation to the attention received by professionals, older patients especially those over 65 years of age had a lower degree of satisfaction, which may be explained by difficulties of aging people in the understanding of therapeutic schedules and a potential reluctance to change. This finding is consistent with other studies that have also reported lower levels of patient satisfaction associated with older age (29, 31). We also found that the period of time during which the patient had previously been collecting OHM at the hospital pharmacy was unrelated to the degree of satisfaction. This result indicates that patient satisfaction with the dispensing strategy via community pharmacies was rated positively, regardless of whether a patient has been using the hospital pharmacy services for a short or long period.

There are patient-centered experiences from other countries in which the patient has been given greater responsibility in the management of their OHM treatment, including mail-order systems medications (5, 7). In a study of a mail-order service for refilling prescriptions for medications, a survey conducted in 219 patients at 1 year after implementation of the service showed that 69.4% of patients were highly satisfied and 27.9% were satisfied. Also, the mail-order fees were determined as a fixed rate of $5, regardless of the number of medications, weight of shipment, or delivery location, and were considered to be reasonable priced by the patients (5). In a study of 57 patients that examined the experience of Veterans with HIV using a VA mail-order pharmacy system, about 90% reported never or rarely having errors with the medications they received and 88% felt once the order was placed, it almost always or usually arrived on time; additionally, many Veterans (53%) indicated that more frequent conversations with a pharmacist could help them manage their conditions better (7). To note, home delivery is currently not covered by the Spanish laws, and also, it is important to emphasize that dispensing of OHM through the community pharmacy allows readily access to pharmacist’s attention at all times.

Decentralized chronic medicine distribution to decrease the frequency of drug collection at health care facilities is a further motivation that may influence adherence to chronic medicines. In a study of patients’ preferences for a last kilometer medicine delivery service model in a high-density housing area of the Cape Town Metropole (South Africa), distance from home to the clinic was a significant variable of preference of medicines to be delivered at home (6). However, our country has a network of more than 22,000 community pharmacies^1^, the largest in Europe, greatly facilitating patient access and encompassing the entire OHM dispensing process among pharmacists.

Limitations of the study included on one side the fact that patients using one or other models were selected based on a number of characteristics that determined lack of baseline comparability of the groups; such differences may result in biases toward a population more prone to be satisfied with the new model. However, considering that the criteria for the use of the new model were suited to identify subjects with higher need of close care, this is consistent with an implementation of a set of criteria that ensures an aligned intervention. Besides, the lack of assessment of adherence to chronic medication of the community pharmacy-based OHM delivery system as compared to the hospital only model. Community pharmacists-led interventions based on guided interaction between the pharmacist and patient have shown to increase adherence in patients using rosuvastatin, irbesartan and/or desvenlafaxine in Australia (32), but a comparison of medication adherence between dispensing modes via community or hospital pharmacy has not been reported. The degree of satisfaction with the involvement of community pharmacies in dispensing OHM was not evaluated over time to determine if there were any changes. Although no single standard measure of patient satisfaction is applicable to all pharmacy situations (33), the questionnaire used in the present study was practical in terms of length and complexity.

The lack of availability of a validated satisfaction survey in this area led to develop a new one according to patient profile and items of interest to be measured. As recommended in this situation, the survey was designed with experts in the area of patients in treatment with OHM (34).

In conclusion, dispensing OHM through the community pharmacy with greater accessibility, mainly due to close distance to the patient’s home, was a strategy associated with greater patient satisfaction as compared with OHM collection at the hospital pharmacy service. The participation of community pharmacists could further optimize the care received by patients undergoing OHM treatment.

## Data availability statement

The raw data supporting the conclusions of this article will be made available by the authors, without undue reservation.

## Author contributions

OFe: Conceptualization, Investigation, Methodology, Project administration, Supervision, Validation, Writing – original draft, Writing – review & editing. SG: Conceptualization, Investigation, Methodology, Project administration, Supervision, Validation, Writing – original draft, Writing – review & editing. EC-G: Data curation, Writing – review & editing. MN-R: Data curation, Writing – review & editing. NC-M: Data curation, Writing – review & editing. MC: Data curation, Writing – review & editing. XF-S: Data curation, Writing – review & editing. LR-N: Data curation, Writing – review & editing. OFa: Writing – review & editing. MB: Writing – review & editing. SP: Writing – review & editing. NT: Writing – review & editing. XD-J: Formal analysis, Methodology, Writing – original draft. CR-C: Writing – review & editing. JC-S: Writing – review & editing. FH: Software, Writing – review & editing. CP-G: Writing – review & editing.

## Footnotes

^1^https://www.farmaceuticos.com/noticias/espana-cuenta-con-la-red-de-farmacias-comunitarias-mas-grande-de-europa/
