# Supplementary material for: Dispensation of outpatient hospital medicines by hospital only versus hospital-community pharmacies collaboration: a cross-sectional study and survey of patient’s satisfaction
Source: Front Public Health. 2024 May 3;12:1335265. doi: 10.3389/fpubh.2024.1335265 (PMC11110910; doi:10.3389/fpubh.2024.1335265)
Supplement: Supplementary file 1 [file Data_Sheet_1.pdf]

Supplementary material

Satisfaction survey to assess the patient's experience in dispensing of outpatient hospital medicines (OHM) via community pharmacies compared to conventional dispensing by the hospital pharmacy service

A. General

1. Sex

- Men
- Women

2. Age

3. How long have you been on treatment with the medication that you have to pick up at the hospital?

- Less than 1 year
- Between 1 and 5 years
- Between 6 and 10 years
- More than 10 years

4. How do you usually collect your OHM?

- I have always gone to pick up the medication at the hospital pharmacy
- Before I went to the hospital, but now I pick it up at the community pharmacy
- Someone else goes to the hospital on my behalf
- They always administered it to me at the hospital

B. If you answer “I have always gone to pick up the medication at the hospital pharmacy” or “Someone else goes to the hospital on my behalf”

5. Waiting time for the collection of medication at the hospital pharmacy:

- Very satisfied
- Satisfied
- Neither satisfied nor dissatisfied
- Dissatisfied
- Very dissatisfied

6. Quality of attention received by personnel of the hospital pharmacy:

- Very satisfied
- Satisfied
- Neither satisfied nor dissatisfied
- Dissatisfied
- Very dissatisfied

7. Information on treatment received by personnel of the hospital pharmacy:

- Very satisfied
- Satisfied
- Neither satisfied nor dissatisfied
- Dissatisfied
- Very dissatisfied

8. Confidentiality in the hospital pharmacy setting:

- Very satisfied
- Satisfied
- Neither satisfied nor dissatisfied
- Dissatisfied
- Very dissatisfied

9. If you want you can leave your comments about the service that are not reflected in the survey.

C. If you answer "Before I went to the hospital, but now I pick it up at the community pharmacy".

10. Waiting time for the collection of medication at the community pharmacy:

- Very satisfied
- Satisfied
- Neither satisfied nor dissatisfied
- Dissatisfied
- Very dissatisfied

11. Quality of attention received by personnel of the community pharmacy:

- Very satisfied
- Satisfied
- Neither satisfied nor dissatisfied
- Dissatisfied
- Very dissatisfied

12. Information on treatment received by personnel of the community pharmacy:

- Very satisfied

- Satisfied
- Neither satisfied nor dissatisfied
- Dissatisfied
- Very dissatisfied

13. Confidentiality in the community pharmacy setting:

- Very satisfied
- Satisfied
- Neither satisfied nor dissatisfied
- Dissatisfied
- Very dissatisfied

14. If you want you can leave your comments about the service that are not reflected in the survey.

Thank you for your cooperation.
